# Mobile genetic elements: the hidden puppet masters underlying infant gut microbiome assembly?

**DOI:** 10.20517/mrr.2024.51

**Published:** 2024-11-09

**Authors:** Kim Kreuze, Ville-Petri Friman, Tommi Vatanen

**Affiliations:** ^1^Institute of Biotechnology, Helsinki Institute of Life Science, University of Helsinki, Helsinki FI-00014, Finland.; ^2^Department of Microbiology, Faculty of Agriculture and Forestry, University of Helsinki, Helsinki FI-00014, Finland.; ^3^Research Program for Clinical and Molecular Metabolism, Faculty of Medicine, University of Helsinki, Helsinki FI-00014, Finland.; ^4^Broad Institute of MIT and Harvard, Cambridge, MA 02142, USA.; ^5^Liggins Institute, University of Auckland, Auckland 1142, New Zealand.

**Keywords:** Colonization bottleneck, MGEs, infant gut microbiome, horizontal transmission, phage, phage-plasmid, plasmid

## Abstract

The gut microbiota is important for healthy infant development. Part of the initial colonizing microbial strains originate from the maternal gut, and undergo a selective event, termed the “colonization bottleneck”. While vertical mother-to-infant inheritance and subsequent colonization of bacteria have previously been studied, the role of mobile genetic elements (MGEs) in the infant gut microbiota assembly is unclear. In this perspective article, we discuss how horizontally and vertically transmitted phages and conjugative elements potentially have important roles in infant gut microbiota assembly and colonization through parasitic and mutualistic interactions with their bacterial hosts. While some of these MGEs are likely to be detrimental to their host survival, in other contexts, they may help bacteria colonize new niches, antagonize other bacteria, or protect themselves from other parasitic MGEs in the infant gut. As a result, the horizontal transfer of MGEs likely occurs at high rates in the infant gut, contributing to gene transfer between bacteria and affecting which bacteria can pass the colonization bottleneck. We conclude by highlighting the potential *in silico*, *in vitro*, and *in vivo* methodological approaches that could be employed to study the transmission and colonization dynamics of MGEs and bacteria in the infant gut.

## INTRODUCTION

**Figure 1:**
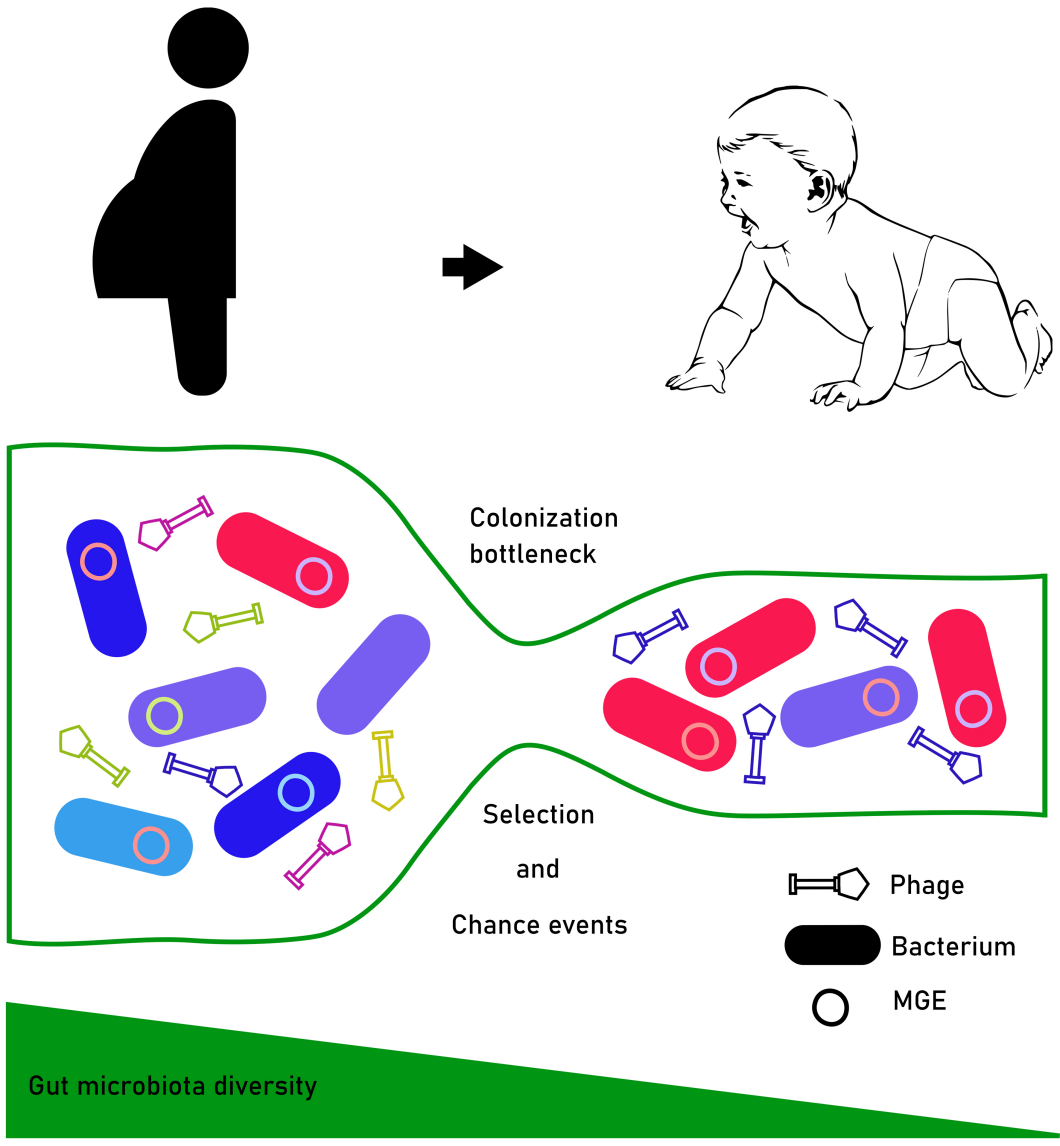
A schematic illustration of the colonization bottleneck and its effect on the diversity and abundance of bacteria and MGEs inherited from mother to infant. Only a subset of bacteria and associated MGEs might be able to pass the colonization bottleneck due to selection and stochastic chance events. The different bacterial taxa and MGEs are represented by different colors and symbols. MGEs: Mobile genetic elements.

The human gut microbiota is a complex community of microorganisms, of which the most predominant and most studied are the bacteria. In the infant gut, this microbiota is vital for infant healthy development as it plays a part in immune system development, energy metabolism, and exclusion of pathogenic microbes^[1-8]^. Development of the microbiota starts at birth when the infants are first exposed to a wide diversity of bacteria from their mothers and the surrounding environment^[9,10]^. This initial community goes through a colonization bottleneck as the population starts at low diversity, with only a subset of bacterial taxa from the mother being vertically inherited to the infant gut^[11-13]^ [Figure 1]. Although the specific ecological processes influencing this colonization event are not entirely understood, prior work suggests that bacterial competition and environmental factors such as oxygen concentrations affect bacterial colonization in infants^[14-16]^. While these advances are important in understanding the assembly of the infant gut microbiota, the influence of mobile genetic elements (MGEs) carried by colonizing bacteria, including plasmids and bacteriophages (phages for short)^[17,18]^, on infant gut microbiome development has been rarely studied^[19-21]^.

In general, MGEs contribute to variable accessory genomes of bacteria and can drive horizontal gene transfer and recombination between different bacterial strains at the population level^[22]^. In the context of crossing the colonization bottleneck, MGEs could help their bacterial hosts adapt and survive by encoding fitness-enhancing genes. On the other hand, MGEs, especially phages, can be antagonistic to bacteria, preventing the colonization of bacteria by lysing them. Furthermore, MGEs often have the potential to move between different bacteria, which could create conflict between different MGEs within the same bacterial cells.

In this perspective, we will first define and examine the colonization bottleneck in the context of infant gut microbiome assembly, explaining how it might impact the vertical inheritance and subsequent colonization of three types of maternal MGEs: phages, conjugative elements, and phage plasmids (P-Ps). We then theorize how different MGEs affect their transmission dynamics and bacterial gene content and how they interact with their host bacteria and other MGE within the infant microbiome. Finally, we highlight the challenges of studying MGEs in gut microbiomes and suggest methodological tools and approaches to tackle these complex interactions in the future.

## INFANT GUT MICROBIOME ASSEMBLY IS AFFECTED BY EARLY COLONIZATION BOTTLENECK

In ecology, a bottleneck is a loss in diversity caused by a large reduction in population size, as seen during the microbial colonization of the infant gut. The low population size makes the infant gut subject to chance events observed, such as when several bacterial strains are either vertically inherited at birth or acquired later on^[11,12]^. However, the population is also subject to unique selection pressures of the infant gut. For example, the relatively high oxygen concentration in the neonatal gut favors facultative anaerobes, such as *Escherichia coli* and *Enterococcus faecalis*^[14,23]^. Additionally, the complex sugars unique to breast milk (human milk oligosaccharides, HMOs) select for bacterial species that metabolize them, such as *Bifidobacterium longum subsp. infantis*^[24]^.

**Figure 2:**
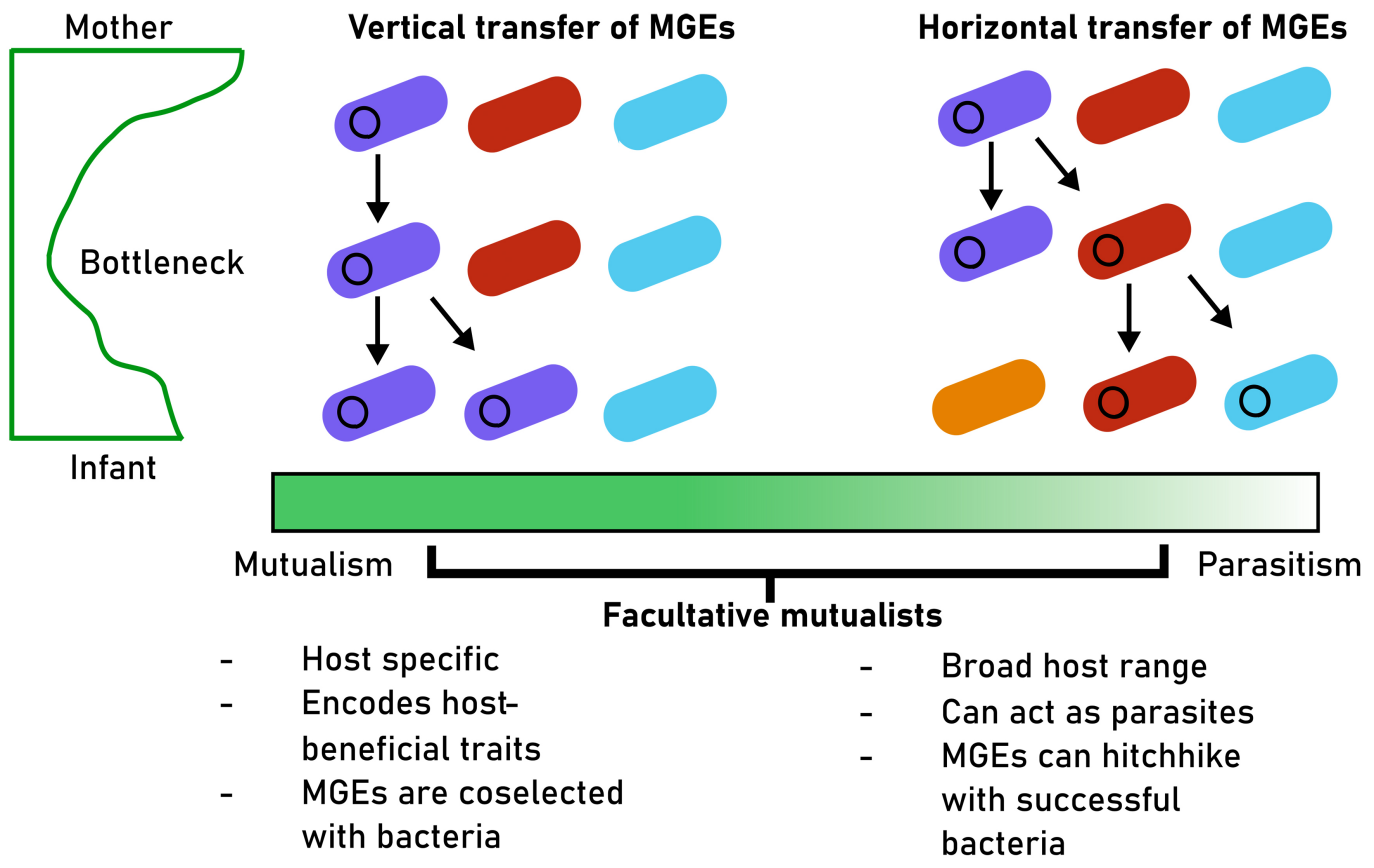
Two alternative routes of MGE inheritance enable them to cross the colonization bottleneck. In the vertical transmission scenario, MGEs are inherited together with their bacterial host during the cell division and this relationship could be beneficial for both (mutualism). In the horizontal transmission scenario, MGEs could move to any successfully colonizing bacterial host, breaking taxonomic associations between the bacterial hosts and their MGEs. This relationship could be considered parasitic as the fitness interests of MGEs are not aligned with those of their host. Most MGEs are capable of both modes of transmission and could hence be considered as facultative mutualists (or parasites)^[25,26]^. MGE: Mobile genetic element.

While MGEs are intimately dependent on their hosts for survival, an MGE can employ two different lifestyles to colonize the infant gut: horizontal or vertical transfer between hosts [Figure 2]. In active horizontal transfer through conjugation or lysis, the MGE may hitchhike on any bacteria successfully passing the bottleneck. Alternatively, an MGE can rely on vertical transfer along with their bacterial hosts^[25,26]^. This can be driven by co-selection, where MGEs that reside in bacterial cells are vertically transferred to the daughter bacterial cells upon cell division along with colonizing bacteria. Very little is known about how common these two strategies are for MGE colonization. Nevertheless, they are likely to have significant consequences for the infant gut microbiome assembly as they provide different fitness benefits and create selection pressures on their host bacteria.

In the case of MGEs that use horizontal transfer to pass the colonization bottleneck, the fitness interests of the original host and MGE are not aligned, as an MGE may survive by hitchhiking onto a different host. It has been proposed that such relationships could be considered “selfish” or parasitic as the MGE would be selected for better transmission at the expense of its host^[27-29]^. Specifically, polyvalent phages that infect a range of bacterial taxa should be favored during the infant gut colonization, given the high and partly stochastic bacterial turnover increases the benefit of their ability to infect multiple hosts^[11,13,30]^. Consequently, horizontally transmitted MGEs could increase their own success in crossing the colonization bottleneck. However, this success can be constrained by the fitness costs they impose on their hosts, as seen with phages, which tend to spread after lysing their bacterial hosts^[31]^.

If an MGE were to transfer vertically along with a bacterial host, we would expect to see co-selection where the host and MGE lineages persist together when transmitted from mothers to infants [Figure 2]. This could be enforced by “mutualistic” MGEs, which provide hosts with genes that improve their fitness^[32-35]^. However, MGEs often create genetic baggage, incurring metabolic costs that render their hosts less fit than the strains that do not carry MGEs^[26,36]^. Consequently, MGEs that primarily transfer vertically are expected to incur low fitness costs and/or compensate for them by providing fitness-enhancing genes to their hosts.

**Figure 3:**
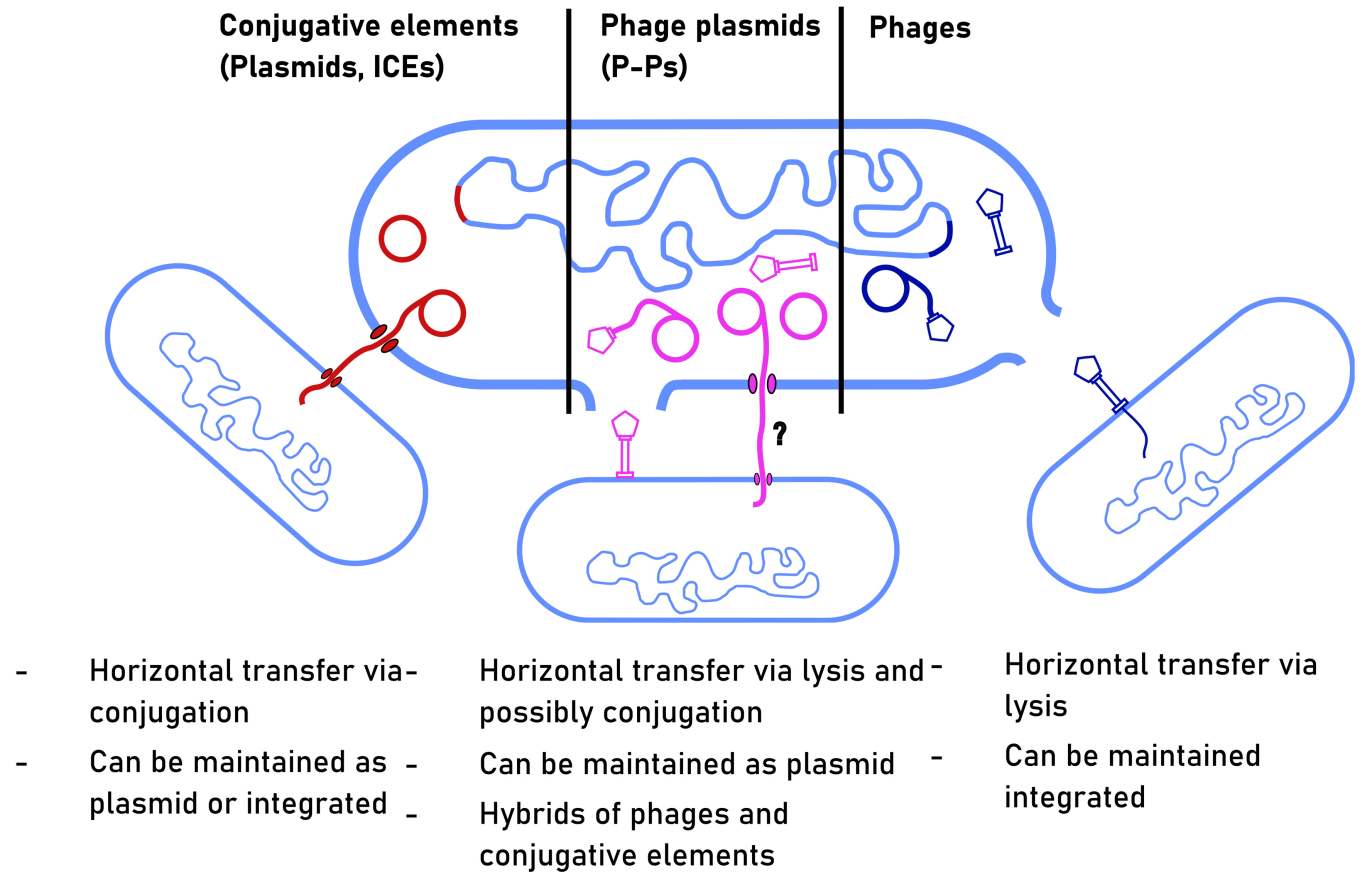
Three types of MGEs and how they are transmitted vertically or horizontally between bacterial cells that are inherited from the mother. The red color (left) represents conjugative elements (e.g., plasmids and ICEs), which can be maintained as extrachromosomal elements or integrated into the host genome and be transferred horizontally between bacterial cells via conjugation. The magenta color (center) represents P-Ps, which can be maintained as plasmids in host cells and transferred via viral lysis and potentially by conjugation. The dark blue color (right) represents phages that can be maintained integrated in the host genome or transferred between bacterial cells by viral lysis. MGEs: Mobile genetic elements; ICEs: integrative and conjugative elements; P-Ps: phage plasmids.

While vertical and horizontal transmission are considered important drivers in the parasitism-mutualist continuum, most MGEs likely fall between these two extremes with the ability to move horizontally or vertically depending on environmental conditions^[25,26]^. Moreover, obligate lytic phages could transmit along with their hosts when in pseudolysogeny, which is a poorly defined state where phages are temporarily dormant in the infected bacterium^[31]^. From this perspective, most MGEs can be seen as facultative mutualists, capable of both vertical and horizontal transmission between bacteria during colonization, which might be beneficial in highly dynamic and unpredictable environments such as the infant gut. We will next present current evidence of how the colonization bottleneck could select three groups of MGEs that are capable of vertical and horizontal transmission between bacterial cells: phages, conjugative elements, and P-Ps [Figure 3].

## A PORTION OF PHAGES, CONJUGATIVE ELEMENTS AND P-PS ARE MATERNALLY INHERITED

Phages are bacterial viruses and the most abundant biological entity on earth^[37]^. Most phages are either obligately lytic or temperate. The lifestyle of obligately lytic phages involves infection of the host cell, production of new viral particles, lysis of the host cell, and release of new viral progeny [Figure 3]. In contrast, a temperate phage can additionally maintain its DNA integrated within its host’s genome as a prophage and transmit vertically to daughter bacterial cells^[31]^. Both phage types can move genetic material horizontally between bacteria, facilitating the transfer of potentially beneficial accessory genes.

Overall, phage and bacterial mother-to-infant inheritance follow similar patterns. Specifically, infant phage richness and diversity are lower compared to the maternal gut, and the infant gut phage turnover rate is extremely high^[25,38,39]^, demonstrating how phages also pass the colonization bottleneck. In addition, approximately 15%-30% of the infant bacteriophage community (phageome) is composed of phages from the mother’s gut^[40,41]^. It is currently thought that most of these phages initially colonize the infant gut as prophages that enter the gut with their hosts^[25]^. There is increased prophage activity throughout infancy that peaks at 1-8 months after birth, with some studies predicting that over 75% of the free phages in the stool are temperate phages^[41-45]^. This induction spike may be an adaptation where phages attempt to establish themselves after the colonization bottleneck by transferring onto more fit hosts, especially given the high bacterial turnover during infancy. Supporting this, Vatanen *et al.* demonstrated that a maternal prophage was found in a different bacterial genome in their infant’s gut^[19]^. Importantly, such dynamics may increase the rate of horizontal gene transfer in the infant gut, as they transfer potential fitness-enhancing genes through processes such as lateral transduction^[46]^.

Conjugative elements are well-studied MGEs composed of integrative and conjugative elements (ICEs, or conjugative transposons) and conjugative plasmids, which both move horizontally between bacterial species through conjugation [Figure 3]. This transfer mechanism does not depend on the lysis of their bacterial hosts but requires close contact between bacterial cells. One key difference between ICEs and plasmids is that the former are typically maintained as integrated into the host chromosome, while the latter are maintained as extrachromosomal elements in the cytoplasm.

Plasmid and ICE compositions tend to be more similar between familial mother-infant pairs and differ between vaginally and Cesarean section delivered infants^[21,47,48]^. For example, a highly prevalent plasmid called pBI143 is frequently identical between mothers and their infants, suggestive of mother-to-infant transmission^[49]^. While studies on conjugation activity in the infant gut are lacking, plasmid groups that are maintained for longer in the infant gut tend to encode conjugation-related genes^[21]^. In addition to this, some conjugative elements have been shown to increase conjugation rate in response to reactive oxygen species and/or the SOS response^[50-53]^, signals that also trigger phage induction^[54]^. The conjugation rate can even be regulated by phage induction itself^[33]^. It is, therefore, possible that conjugative elements and phages may cross the colonization bottleneck by promoting their own conjugation and horizontal transfer.

P-Ps are hybrids between the MGEs they are named after, as they tend to encode both phage and plasmid genes^[55-57]^. While their diversity is only beginning to be uncovered, they mostly follow a lifestyle similar to temperate phages: they can enter lytic or temperate life cycle, but differ in that as a prophage, they form an extrachromosomal plasmid. While cultured P-Ps are only known to transfer horizontally through lysis, similar to phages, a subset of P-Ps encode genes necessary for conjugation and may therefore be able to transmit via conjugation [Figure 3].

Even though the abundance and diversity of P-Ps have only recently been studied^[55,58]^, evidence suggests that infant gut P-Ps might also be maternally inherited. First, P1-like P-Ps, relatives of the well-studied P1 phage^[59]^, were found in clinical and porcine-derived *E. coli* isolates, with the latter being isolated from the stool of a healthy 4-month-old piglet^[60,61]^. Additionally, some of the plasmids predicted to be N15-like P-Ps, relatives of the well-studied N15 phage^[62]^, were isolated from bacterial hosts in dogs, giant pandas, and humans^[56]^. Finally, at least a fraction of phages of the family Crassvirales, the most abundant phage family in the human gut, appears to be P-P^[63]^ and members of this phage family can be transmitted from mother to child^[64]^. While this evidence is intriguing, targeted studies confirming the presence and transmission of P-Ps between the mother and infant guts are required.

High bacterial turnover, stress, and phage induction levels suggest that MGEs can transmit horizontally between bacteria during infant gut colonization. While such dynamics may be necessary for MGEs in crossing the colonization bottleneck, they may also come at a fitness cost for their bacterial hosts, given that MGE spread, excess conjugation, and phage lysis can inhibit bacterial growth^[31,65]^, emphasizing a parasitic interaction. However, such fitness costs are dependent on the ecological context. For example, while conjugative plasmids and phages can be costly to maintain, they can also provide fitness benefits to their bacterial hosts even in the absence of obvious selection pressures^[66-68]^. Most commonly, however, MGEs are thought to be maintained by carrying genes that can increase the fitness of their hosts^[68]^. In the next section, we explore the fitness-enhancing genes carried by MGEs and investigate how they could aid bacterial hosts in crossing the colonization bottleneck.

## MGES CONTRIBUTE TO BACTERIAL COLONIZATION SUCCESS IN THE INFANT GUT

**Figure 4:**
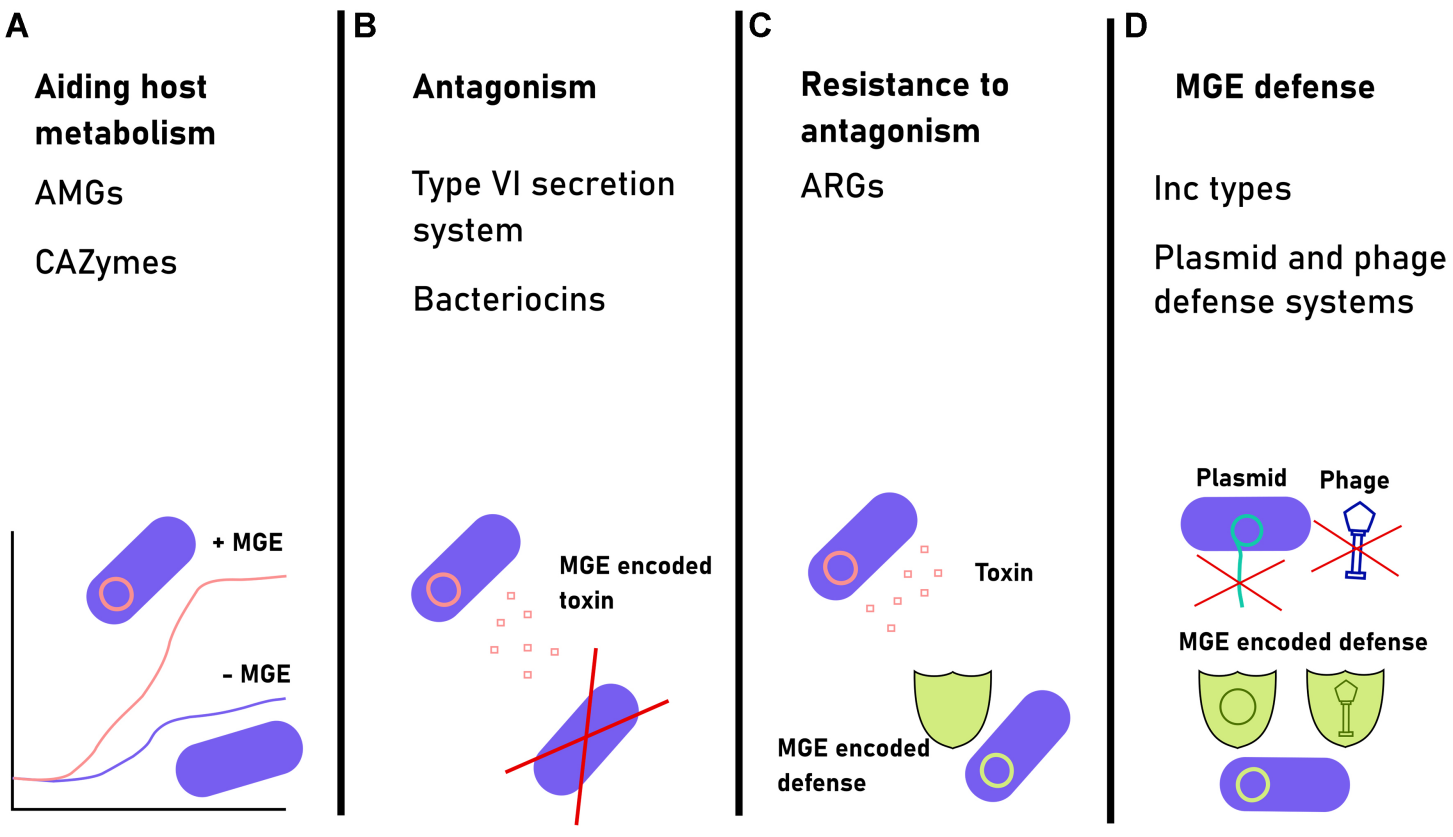
Range of mechanisms of how the MGE-encoded beneficial functions might help their host bacteria to cross the colonization bottleneck. These functions include (A) aiding host metabolism and growth by encoding genes such as AMGs and CAZymes; (B) antagonizing competitor bacteria by encoding type VI secretion systems and bacteriocins; (C) resisting antibiosis competition by encoding ARGs, such as reuterin resistance genes^[69]^; and (D) defending the host from parasitic MGEs through Inc types, superinfection exclusion, and plasmid and phage defense systems. MGE: Mobile genetic element; AMGs: auxiliary metabolic genes; CAZymes: carbohydrate-active enzymes; ARGs: antibiotic resistance genes.

Some bacterial taxa only temporarily colonize the sparsely seeded infant gut and are subsequently outcompeted by other bacterial taxa^[11,13,30]^. Thus, the ability to aid in host metabolism, inhibit competitor growth, and protect oneself from competitors and parasites is important in influencing the successful crossing of the colonization bottleneck [Figure 4]^[69]^. Fitness-enhancing genes providing such functions can be encoded by MGEs and could, therefore, be co-selected with their hosts.

While direct evidence of MGEs aiding their hosts to increase their metabolic capacity through novel genes is lacking, metagenomic studies provide some tentative evidence. Specifically, prophages in the infant gut encode several auxiliary metabolic genes (AMGs)^[70]^, including carbohydrate-active enzymes (CAZymes) related to carbon, amino acid, and energy metabolism^[71-74]^ [Figure 4A]. These genes are likely involved in processes such as HMO and mucin attachment and degradation, as well as increasing growth rate by encoding more copies of rate-limiting genes^[75]^. Regarding conjugative elements, a recent study found that plasmids in the infant gut encode proportionally more unique metabolism-related genes compared to their bacterial hosts^[21]^. Additional bioinformatic and functional evidence suggests that conjugative elements can provide metabolic benefits to bacterial strains found in the gut microbiome^[18,35,76-78]^. In addition to this, most phages and plasmids encode a wide range of hypothetical proteins with no known functions, which could potentially provide important, novel functions to their hosts.

Besides aiding their hosts to increase their metabolic potential, MGEs could help their hosts colonize the infant gut by antagonizing or protecting against competitor bacteria. Specifically, phages and conjugative elements have been observed to carry bacteriocins and different types of growth-inhibiting genes which can provide a competitive edge against other bacterial strains^[34,79-84]^ [Figure 4B]. Such genes have been suggested to be important for bacterial infant gut colonization^[16,85]^ and may promote the transfer of MGEs, as in the case of *Pseudomonas aeruginosa* phages in an insect model^[86]^. Antibiotic resistance genes (ARGs) are another example of well-known genes that provide defense against competitor bacteria [Figure 4C]. In support of their importance, the infant gut harbors more ARGs compared to adults^[48,87]^, and these are mostly carried by conjugative elements but not phages^[88]^. Additionally, P-Ps carry ARGs more often than phages, which indicates that they may contribute to the gut resistome^[56]^. More research is hence needed to explore if ARG carriage in MGEs provides a colonization benefit in the infant gut due to antibiotic usage or competition between other bacteria.

Finally, the fitness costs of the increased MGE horizontal transfer during the induction spike could constrain the prevalence of MGEs such as phages. Indeed, a few studies have proposed that the infant phageome follows the kill-the-winner (KtW) or kill-the-competitor (KtC) population dynamics as a result of selective phage predation targeting a subset of bacterial taxa^[89,90]^. Perhaps counterintuitively, various phage types, such as cryptic prophages, have been shown to encode an enormous diversity of functional phage defense genes that protect their bacterial hosts from both closely related and highly distinct phages^[91-97]^. Several of these defense systems can also target plasmids^[93,96]^ [Figure 4D]. Although many defense systems seem to be exclusively encoded in phage genomes, Restriction-Modification (R-M), Toxin-Antitoxin (T-A), and clustered regularly interspaced short palindromic repeats (CRISPR) systems, for example, are also encoded in conjugative elements suggesting that to inhibit phage replication^[33,98-102]^. Plasmids can also exclude other related plasmids based on incompatibility (inc) types^[47,103]^. Although inc types are not traditionally thought of as a form of MGE defense, plasmid populations can be driven into extinction due to incompatibility^[104,105]^. It is, therefore, plausible that P-Ps have the greatest genetic potential in MGE defense since they can both exclude plasmids of the same inc type and also potentially protect bacteria against phages^[106,107]^.

## CURRENT CHALLENGES AND SOLUTIONS

While accumulating evidence suggests that MGEs can have a profound influence on the bacterial colonization of the infant gut, deciphering the roles and significance of different MGEs is challenging due to the inherent genomic characteristics of MGEs. First, the genome mosaicism and large amount of unknown open reading frames make MGEs and their functions difficult to quantify and characterize in metagenomic datasets. In particular, robust methodologies to identify and characterize novel P-Ps are urgently needed. Second, it is technically difficult to ascertain a host for an MGE in metagenomic datasets if the MGE is integrated into multiple sites or exists extrachromosomally, which is required for tracking its vertical and horizontal transmission between bacterial taxa.

While finding such signals in metagenomic datasets is challenging, promising sequencing and bioinformatic approaches are emerging. First, long-read sequencing technologies, such as PacBio and Oxford Nanopore Technologies (ONT), provide reads long enough to cover both an MGE and an associated bacterial genome. Additionally, ONT can provide an adaptive sequencing option that could be leveraged to selectively deplete non-MGE DNA or amplify MGEs that are at low abundance^[108]^. Using state-of-the-art bioinformatic tools combining machine learning and homology-based approaches, such as PlasX, VirSorter, VirFinder, and geNomad, to identify MGEs can also be used^[18,109-111]^.

While these technologies enable the identification of several groups of MGEs, establishing the physical association of extrachromosomal MGEs with their hosts remains challenging. A technique called Hi-C, which involves fixing and cross-linking DNA molecules with proximity (both chromosomal and extrachromosomal DNA) followed by sequencing, could be used to resolve this. Moreover, microfluidics and single-cell sequencing have been used to successfully link extracellular elements, such as phages undergoing lytic infection and plasmids, to their hosts^[112-114]^.

Unfortunately, the abovementioned techniques do not provide direct evidence for the identification of P-Ps. For example, *Carjivirus communis* phage was only recently confirmed to be a P-P through rigorous experimentation^[63]^. Thus, culturing remains a highly relevant technique for identifying and characterizing MGEs, as isolating gut bacteria, phages, and other MGEs enables direct experiments in lab cultures and the creation of synthetic model bacterial communities^[115,116]^, which could be used to unravel the ecological and evolutionary roles of MGEs in model infant gut microbiomes. Finally, organoid and *in vivo* animal models with humanized gut microbiomes could provide opportunities to incorporate host cells and immune systems with controlled community experiments^[117]^.

While this perspective focused only on a subset of MGEs, further work should also consider other smaller MGEs, such as “hitchers”, which are genetic elements that require other MGEs to transfer horizontally between bacterial hosts^[118]^. For example, phage satellites can lower the amount of phage particles that their helper phage produces upon lytic infection and can encode a wide array of phage defense genes^[119-121]^. To fully understand the impact of MGEs in the infant gut, we must consider the whole mobilome, including hitchers and other smaller MGEs that are incapable of transferring between bacterial cells.

## CONCLUSIONS AND FUTURE PERSPECTIVES

In summary, there is currently a lack of evidence regarding the roles of MGE in infant gut bacterial assembly and colonization. Understanding what MGEs are maternally inherited and the underlying selection pressures and stochastic factors that affect MGE colonization are likely extremely important for understanding the infant gut microbiome assembly. We hypothesize that the stressors involved in crossing the colonization bottleneck are important drivers for MGE movement, which in turn is likely to affect the phylogenetic and functional composition of the bacteriome. Specifically, the temperate phage induction spike in infancy reflects the potential importance of horizontally transmitted MGEs in the infant gut. MGE-encoded accessory genes might lead to co-selection with associated bacteria, while defense systems targeting MGEs might limit their horizontal transmission and recombination. As more research is performed on viral dark matter and undiscovered MGEs, we will start better understanding the potential role of MGEs as puppet masters behind infant gut microbiome assembly.

Future research should focus on developing more robust methodologies for identifying and characterizing MGEs, especially novel P-Ps, in infant metagenomic datasets from diverse geographic regions. This is important to accurately assess the prevalence, abundance, and genetic content of MGEs in different populations. It would also be beneficial to collect more dense data, optimally with daily sampling, to understand early *in vivo* MGE dynamics in the infant gut in higher resolution. Further bioinformatic and experimental evidence into the functional roles of MGE-encoded genes in the infant gut, particularly those involved in metabolism, antagonism, and MGE defense, is required. Additionally, controlled experiments using multi-species synthetic bacterial communities and animal models could help elucidate the impact of environmental stressors on MGE transmission dynamics and the relative fitness benefits and costs they may impart to their hosts. Finally, expanding research to encompass a wider array of MGEs, including smaller elements like “hitchers”, will provide a more comprehensive understanding of the infant gut mobilome and its influence on microbial community assembly.

**Glossary:** Vertical inheritance: The inheritance of microbes or MGEs from mother to child upon birth. Vertical MGE transmission: The transmission of MGEs to the daughter cells of the same bacteria by cell division. Horizontal MGE transmission: The transmission of mobile MGEs between different bacterial cells independent of cell division. MGE/phage defense genes: Genes dedicated to interfering with the replication or maintenance of MGEs or phages that have infected the cell. Colonization bottleneck: A process that limits what microbes can colonize the infant gut microbiome, resulting in a decrease in genetic and taxonomic diversity. Kill-the-winner (KtW) dynamics: A predator-prey type dynamic where phages control populations of the most successful bacteria through lysis. Kill-the-competitor (KtC) dynamics: A predator-prey type dynamic where phages protect their original host bacteria by lysing competitive bacteria. Core genome: The part of the bacterial genome that is conserved in all strains within the same taxa. Accessory genome: The variable part of the bacterial genome that is not found in all strains within the same taxon.

## DECLARATIONS

### Copyright

© The Author(s) 2024.
